# Using swin UNETR deep model for automated detection of alveolar bone fenestration/dehiscence in CBCT

**DOI:** 10.3389/fbioe.2026.1752350

**Published:** 2026-02-12

**Authors:** Ailin Xu, Hanxiao Huang, Bin Zhang, Shan Dong, Xiaoxia Che

**Affiliations:** 1 Department of Orthodontics, Beijing Stomatological Hospital, Capital Medical University, Capital Medical University School of Stomatology, Beijing, China; 2 MeiQi Technology, Hangzhou, Zhejiang, China

**Keywords:** cone beam computed tomography, deep learning, dehiscence, fenestration, SwinUNETR

## Abstract

**Objectives:**

This study aims to develop a deep learning-based model for the automatic detection of fenestration and dehiscence in Cone Beam Computed Tomography (CBCT) images, providing a quantitative tool for diagnosing alveolar bone defects.

**Methods:**

Utilizing 10,752 manually annotated sagittal CBCT dental images, the Shifted Window Transformer U-Net (Swin UNETR) model was trained to automatically measure and diagnose fenestration and dehiscence. Model performance was evaluated based on key point localization accuracy, length measurement accuracy, and disease detection performance. Heatmaps were employed for visual identification of disease locations.

**Results:**

The Swin UNETR model achieved key point recognition rates of 92.97%–99.09% for fenestration and dehiscence. Predicted lengths for all defect sites showed strong correlation with actual measurements. Disease diagnosis accuracy ranged from 0.8228 to 0.9476. The model demonstrated robust performance in key point identification, defect length quantification, and disease diagnosis.

**Conclusion:**

The deep learning model enables precise localization and quantitative measurement of fenestration and dehiscence in CBCT images. This approach enhances diagnostic efficiency and accuracy in detecting fenestration and dehiscence, facilitating preoperative orthodontic risk assessment and personalized treatment planning.

## 1 Introduction

Dehiscence is diagnosed when an alveolar bone defect involves the alveolar bone crest and forms a V-shaped defect with the cementoenamel junction (CEJ). Fenestration is diagnosed when an alveolar bone defect occurs apical to the alveolar crest, interrupting the continuity between the alveolar crest and the root apex (Sun et al., 2015). Thes e two primary forms of alveolar bone defects, Dehiscence and Fenestration, are significant risk factors for gingival recession and root resorption, influencing orthodontic treatment planning and outcomes (Luo et al., 2024).

Previous studies on alveolar bone defects primarily utilized dry skulls, periodontal flap surgery, and radiographic examinations (Leung et al., 2010; Alsino et al., 2022; Han et al., 2024). Among various radiological modalities, cone beam computed tomography (CBCT) has demonstrated the highest sensitivity and diagnostic accuracy in detecting various periodontal defects (Bagis et al., 2015). While clinical surgical exposure remains the gold standard for diagnosing bone fenestration and dehiscence, CBCT is preferred for its non-invasive nature, convenience, and reliability (Peterson et al., 2018; Ruetters et al., 2022). CBCT demonstrates 100% sensitivity in detecting alveolar bone defects. The specificity ranges from 45.5% to 86.7% for Dehiscence and 50%–86.7% for Fenestration, indicating relatively higher false-positive rates. Nonetheless, CBCT is widely recognized as a reasonably acceptable tool for detecting these defects (Yagci et al., 2012; Alsino et al., 2022). CBCT with voxel sizes ≤0.4 mm is a reliable instrument for linear measurements (Patcas et al., 2012), and smaller voxel sizes combined with a smaller field of view yield the highest sensitivity and diagnostic accuracy (Icen et al., 2020).

Current diagnostic criteria for alveolar bone defects typically require the absence of cortical bone surrounding the tooth root across at least three consecutive sagittal views (Cha et al., 2021). The positive threshold for dehiscence is generally defined as a defect depth ≥2 mm, while fenestration is diagnosed based on the interruption of bone continuity, irrespective of defect size (Evangelista, Vasconcelos Kde et al., 2010; Sun et al., 2015; Han et al., 2024). The strictest criteria define fenestration at a 2.2 mm threshold (Sun et al., 2022). Consequently, CBCT has become the clinically preferred method for evaluating dehiscence and fenestration due to its non-invasive nature. However, its diagnostic accuracy remains highly dependent on manual image interpretation, a process inherently subjective and whose efficiency varies significantly with operator experience.

With the rapid advancement of deep learning technology in medical image analysis (Liu et al., 2025), convolutional neural networks (CNNs), Transformer architectures, and YOLO models have become core technologies for intelligent diagnosis in oral and maxillofacial imaging (Beser et al., 2024; Vilcapoma, Parra Meléndez et al., 2024; Ramírez-Pedraza et al., 2025). CNNs extract local features through hierarchical convolutional kernels and utilize translation invariance to accomplish image classification and segmentation tasks. Their classic architectures, such as U-Net and ResNet, have shown significant progress in anatomical structure detection, dental disease diagnosis, and periodontal disease assessment (Chang et al., 2020; Liu et al., 2022; Asgary, 2024; Zhu et al., 2024). Transformers, based on the self-attention mechanism, enhance feature representation capabilities by globally modeling long-range dependencies. They have achieved significant success in computer vision tasks (Zhou et al., 2024).

Swin UNETR (Shifted Window Transformer U-Net) (Kakavand et al., 2024) effectively integrates the strengths of both paradigms. It employs a hierarchical shifted window mechanism to achieve cross-scale feature interaction while significantly reducing the computational burden inherent in standard Transformers. Furthermore, by incorporating the U-Net encoder-decoder architecture, it preserves crucial spatial details, thereby providing a novel approach for identifying subtle lesions and modeling complex anatomical structures. Applying deep learning models for disease diagnosis can mitigate the influence of subjective judgment and variability, shorten diagnostic and treatment timelines, and enhance diagnostic accuracy and therapeutic efficiency (Sistaninejhad et al., 2023). Critically, no published studies have yet reported the application of Swin UNETR for imaging-based detection of alveolar bone defects in the oral cavity.

To enhance annotation accuracy and reliability, the present study opted to perform annotations on two-dimensional sagittal section images derived from CBCT data. This approach effectively circumvents the information loss inherent in the three-dimensional reconstruction process, thereby providing a high-quality data foundation for the training of subsequent image recognition models. This study aims to validate the capability of a deep learning system in localizing, measuring, and diagnosing fenestration and dehiscence on CBCT sagittal sections. The primary objective is to provide dental clinicians with an intelligent auxiliary diagnostic tool to improve the efficiency and objectivity of assessing these bone defect conditions.

## 2 Materials and methods

### 2.1 Data acquisition

Written informed consent was obtained from all participants prior to CBCT scanning. The consent forms included permissions for data usage in scientific research and algorithm development. The Ethics Committee approved this research protocol (Approval No. CMUSH-IRB-KJ-PJ-2024-69). Participants were selected from patients with malocclusion who visited the department of orthodontics between January 2021 and December 2023. The inclusion and exclusion criteria are detailed in Table 1. CBCT scans were acquired for all patients using the NewTom VGi system (AFP Imaging, Verona, Italy). Scans were performed using the standard acquisition mode with the following parameters: tube voltage 110 kV, tube current 1–20 mA (automatic exposure control), FOV 15 cm × 15 cm, and voxel size 0.25 mm. During acquisition, the X-ray tube rotated 360° around the patient, with an exposure time of approximately 3.6 s. Image reconstruction was completed in approximately 1 min. All CBCT datasets were exported in Digital Imaging and Communications in Medicine format and processed using NNT Viewer software (v5.6.0.0).

**TABLE 1:** Participant inclusion and exclusion criteria.

Inclusion criteria
(1) Patients aged 13–45 years old, regardless of gender (2) Patients who underwent CBCT scans in the NewTom VGi format
Exclusion criteria
(1) History of maxillofacial trauma (2) History of cysts or tumors (3) History of craniofacial surgery (4) History of cleft lip and palate, craniofacial defect syndrome, or skeletal malformation (5) Presence of retained deciduous teeth, supernumerary teeth, and impacted teeth (6) Medical history of the whole body system (7) The images have motion artifacts and metal artifacts

**FIGURE 1:**
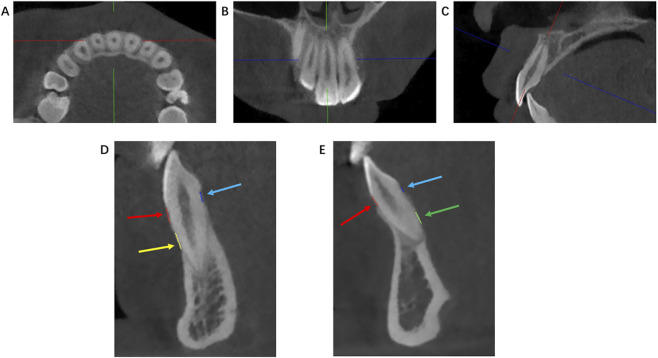
Method to define the measurement plane. **(A)** Transverse view, with the sagittal line (green) passing through the labiopalatal/buccolingual sides. **(B)** Coronal view, with the sagittal line (green) passing through the midpoint between the root apex and the incisal edge/cusp tip. **(C)** Sagittal view, with the coronal line (red) passing through the root apex and the incisal edge/cusp tip. **(D)** The red line indicates the labial/buccal dehiscence length, the blue line indicates the lingual/palatal dehiscence length, the yellow line indicates the labial/buccal fenestration length. **(E)** The green line indicates the lingual/palatal fenestration length, with red and blue denoting the same as in part **(A)**.

The measurement process for fenestration and dehiscence was conducted as follows, this procedure ensured the reproducibility of the measurement plane by employing well-defined anatomical landmarks: (1) Define the measurement plane using the sagittal, coronal, and transverse planes, indicated by green, red, and blue lines respectively. (2) Adjust the horizontal view to capture the maximum cross-section of the root in the labiopalatal/buccolingual direction (Figure 1A). (3) Modify the coronal view so the sagittal line intersects the midpoint between the apex and the incisal edge/cusp tip (Figure 1B). (4) Fine-tune the sagittal view to ensure the coronal line passes through both the apex and the incisal edge/cusp tip (Figure 1C). All images were acquired in the sagittal view. Centered on this reference sagittal plane, a series of three consecutive sagittal images were obtained by offsetting one slice thickness (0.25 mm) each in the mesial and distal directions. All images were captured at 200% magnification and adjusted to a grayscale value of 50%. An evaluator independently assessed these three images. On a single image, any region where the root was not covered by the cortical bone was recorded as an alveolar bone defect for that specific image (Sun et al., 2022). Dehiscence is diagnosed when the alveolar bone defect involves the alveolar crest, and the distance between the bottom of the V-shaped defect and CEJ exceeds 2 mm. Fenestration is diagnosed when the alveolar bone defect does not involve the alveolar crest, and the interruption distance between the alveolar crest and the apex is greater than 2.2 mm (Evangelista, Vasconcelos Kde et al., 2010; Sun et al., 2015). A tooth was diagnosed with a bone defect (fenestration/dehiscence) based on the following comprehensive criterion: a positive diagnosis was assigned only if all three consecutive images consistently showed a positive finding. The result was then recorded accordingly.

The study analyzed 160 CBCT scans, comprising 3,584 teeth (1,558 anterior and 2026 posterior) and 10,752 images (4,674 anterior and 6,078 posterior). Images with dimensions of 320 × 224 pixels were extracted from the sagittal view and imported into the image annotation software Microsoft Paint (11.2405.17.0). Two orthodontists, each with 6 years of clinical experience and expertise in CBCT evaluation, manually marked dehiscence and fenestration locations. To ensure accuracy, images were randomly selected and annotated three times. The two endpoints of dehiscence were the alveolar ridge crest (ARC) and CEJ, while the two endpoints of fenestration were the coronal border (CB) and apical border (AB). For annotation, fenestration and dehiscence were demarcated using line segments in distinct colors. Annotations used color codes: red for labial/buccal dehiscence, yellow for labial/buccal fenestration, blue for lingual/palatal dehiscence, green for lingual/palatal fenestration (Figures 1D,E). Inter-examiner reliability was assessed using the kappa coefficient to ensure diagnostic consistency. For images with discrepant evaluation results, the final determination was made through discussion between the two researchers. If consensus could not be reached, an orthodontic chief physician with 30 years of experience assisted in the evaluation.

### 2.2 Experimental setup

#### 2.2.1 Dataset preparation

To maintain patient-level independence across training, validation, and testing subsets, we used a 7:2:1 split, assigning all slices from each patient to a single set. The training set (6,879 slices) was used for model optimization, the validation set (2,451 slices) for hyperparameter tuning, and the independent testing set (1,422 slices) for final performance evaluation. All images were normalized in grayscale intensity to enhance contrast stability and learning convergence.

To enhance model robustness and mitigate overfitting, the training dataset was augmented using various techniques. Random scaling, rotation, translation, Gaussian blurring, and additive noise were applied to the samples, simulating real-world variability and improving the generalization of the learned representations. All input images were standardized prior to being fed into the model to stabilize the training process and accelerate model convergence.

#### 2.2.2 Model configuration

This study employs a landmark prediction model built on the Swin UNETR architecture, which integrates the Swin Transformer’s representational capabilities with the semantic insights of UNet-based encoder-decoder structures. Swin UNETR utilizes a hierarchical Vision Transformer backbone to encode long-range dependencies and a symmetric decoding path for spatial localization (Kakavand et al., 2024).

**FIGURE 2:**
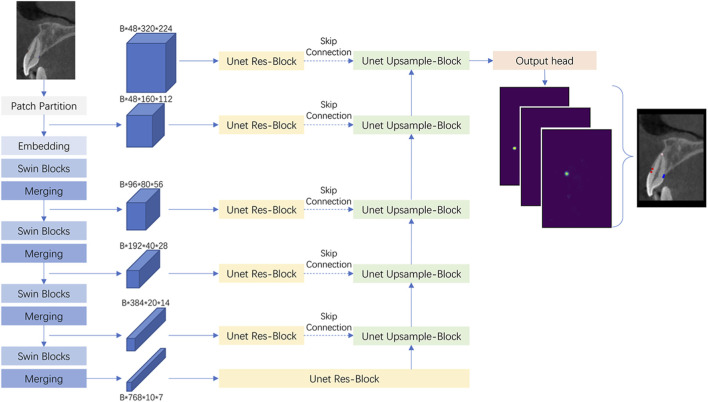
Overview of the swin UNETR architecture.

The encoder consists of multiple Swin Transformer blocks arranged hierarchically across four stages. Each stage partitions the input into non-overlapping windows, and self-attention is computed within each window. This shifted windowing mechanism alternates between layers, enabling cross-window interaction while maintaining computational efficiency. Specifically, the encoder stages produce feature maps at progressively coarser spatial resolutions using patch merging operations. The decoder path uses transposed convolutions and skip connections to recover high-resolution predictions, with multi-level features concatenated from the encoder via residual paths (Figure 2) (Kakavand et al., 2025).

The output of the model is a 12-channel prediction map corresponding to various anatomical targets:

Channels 1–3: Labial/buccal (length/area of dehiscence). Channel 1 predicts a binary segmentation mask of the (length/area of dehiscence) region. Channels 2 and 3 estimate the normalized direction vector $\vec{d}=\left(d_{x},d_{y}\right)$ , roughly in the same direction from the root apex to the crown. Channels 4–6: Lingual/palatal (length/area of dehiscence), structured identically to channels 1–3. Channels 7–9: Labial/buccal (length/area of fenestration). Channels 10–12: Lingual/palatal (length/area of fenestration). As defined by Equation 1, the heatmap h(x, y) is computed as:

$$h\left(x,y\right)=\sum_{i=1}^{k}\exp\left(-\frac{{\left(x-x_{i}\right)}^{2}+{\left(y-y_{i}\right)}^{2}}{2\sigma^{2}}\right)$$ (1)

Where $\left(x_{i},y_{i}\right)$ denotes the coordinates of the i -th tooth root landmark for the given image slice, k is the number of annotated roots, and σ controls the spread of each Gaussian (typically fixed based on voxel resolution). Each of the binary mask channels (i.e., channels 1, 4, 7, and 10) are supervised using binary cross-entropy loss (BCE). All direction vector and heatmap channels are trained with mean squared error (MSE) loss, allowing for continuous-valued regression appropriate for directional and heatmap outputs.

#### 2.2.3 Training details

The model was trained for 200 epochs using the AdamW optimizer with an initial learning rate of 1e-4. A cosine annealing learning rate scheduler was applied to gradually reduce the learning rate over time. All training and evaluation were implemented in PyTorch, and experiments were conducted on a workstation equipped with an NVIDIA RTX 4090 GPU.

### 2.3 Performance evaluation

The model was evaluated on an independent test set through a comprehensive assessment across three dimensions: key point localization, length measurement, and disease detection performance. Key Point Localization was assessed using the point recognition rate and metrics derived from Euclidean distance. The point recognition rate was defined as the proportion of correctly identified key points to the total number of key points. Distance-based evaluation included the average Euclidean distance (AED), standard deviation (SD), and quartiles (first quartile Q1, median, third quartile Q3) to comprehensively evaluate localization accuracy and stability. Length measurement was calculated from the predicted coordinates of two endpoints and compared with manually measured lengths. The evaluation included the mean absolute error (MAE), mean relative error (MRE), root mean squared error (RMSE), Pearson correlation coefficient (PCC) to assess linear correlation between predicted and true lengths, and Bland–Altman analysis with 95% limits of agreement to evaluate the consistency between the two measurement methods. Disease detection performance was benchmarked against manually annotated diagnostic results as the gold standard. Model performance was evaluated at both the single-image level and the tooth level (requiring positivity across three consecutive images). Metrics included accuracy, recall, precision, specificity, F1 score, receiver operating characteristic (ROC) curve, and area under the ROC curve (AUC). These evaluation metrics are computed using Equations 2–6. The detailed formulas for disease detection performance are as follows:

$$\text{Accuracy}=\frac{\;\text{TP}+\text{TN}}{\text{TP}+\text{TN}+\text{FP}+\text{FN}\;}$$ (2)

$$\text{Recall}=\frac{\;\text{TP}}{\text{TP}+\text{FN}\;}$$ (3)

$$\text{Precision}=\frac{\;\text{TP}}{\text{TP}+\text{FP}\;}$$ (4)

$$\text{Specificity}=\frac{\;\text{TN}}{\;\text{TN}+\text{FP}\;}$$ (5)

$$F1=2\times \frac{\text{Precision}\times \text{Recall}}{\text{Precision}+\text{Recall}}$$ (6)

(TP, true positive; TN, true negative; FP, false positive; FN, false negative).

## 3 Results

**FIGURE 3:**
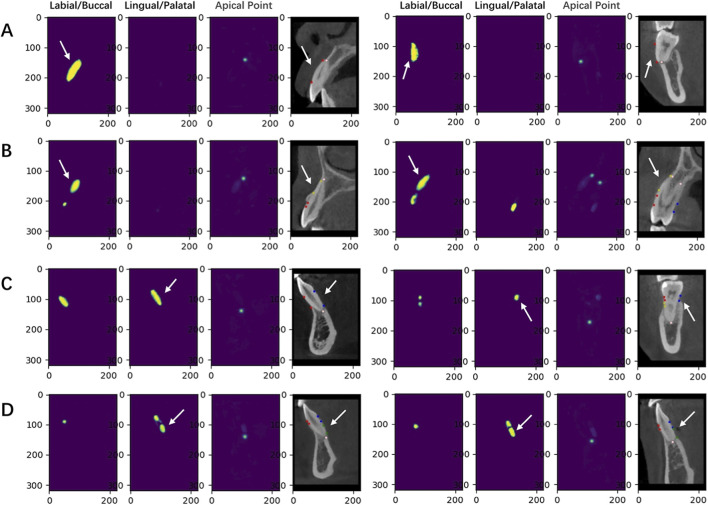
Images of dehiscence and fenestration and its identification effect map. **(A)** Labial/buccal area of dehiscence. **(B)** Labial/buccal area of fenestration. **(C)** Lingual/palatal area of dehiscence. **(D)** Lingual/palatal area of fenestration.

The Kappa value for the consistency test was 0.92. The Swin UNETR model first identified the heatmap range for dehiscence and fenestration, and then performed point recognition at both ends of the bone defect (Figure 3). The point recognition rate for bone defects ranges from 92.97% to 99.09%. The median Euclidean distance errors measured 0.2928–0.3131 mm for dehiscence landmarks, 0.2924–0.4270 mm for fenestration landmarks, as detailed in Table 2.

**TABLE 2:** Key point localization accuracy.

Performance metric	Labial/Buccal dehiscence		Labial/Buccal fenestration		Lingual/Palatal dehiscence		Lingual/Palatal fenestration	
	ARC	CEJ	CB	AB	ARC	CEJ	CB	AB
Recognition rate	92.97%		99.09%		96.91%		97.75%	
AED (mm)	0.3733	0.3537	0.5076	0.4450	0.3697	0.3597	0.4002	0.3733
SD (mm)	0.3127	0.2712	0.3396	0.2816	0.2576	0.2479	0.3368	0.2803
Q1 (mm)	0.1743	0.1866	0.2458	0.2275	0.1929	0.1811	0.1992	0.1819
Median (mm)	0.2954	0.2928	0.4270	0.3820	0.3131	0.3058	0.2971	0.2924
Q3 (mm)	0.4639	0.4512	0.7166	0.6230	0.4711	0.4718	0.4937	0.4972

**FIGURE 4:**
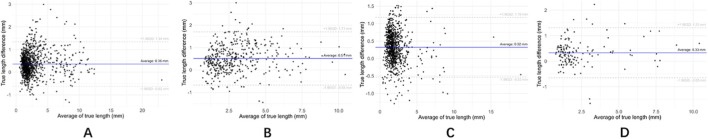
Bland-Altman analysis plot of the predicted length and the true length. **(A)** Labial/buccal dehiscence. **(B)** Labial/buccal fenestration. **(C)** Lingual/palatal dehiscence. **(D)** Lingual/palatal fenestration.

The accuracy of length measurements is presented in Table 3. The mean relative errors for dehiscence are 0.3008 and 0.3550, while for fenestration they are 0.3291 and 0.3134. The Pearson correlation coefficients for labial/buccal dehiscence, labial/buccal fenestration, lingual/palatal dehiscence, and lingual/palatal fenestration were 0.9722, 0.9409, 0.9480, and 0.9625, respectively. The model-predicted lengths showed good correlation with the actual lengths at all four sites, and the errors at these sites fell within the 95% confidence interval (Figure 4).

**TABLE 3:** Length measurement accuracy.

Performance metric	Labial/Buccal dehiscence	Labial/Buccal fenestration	Lingual/Palatal dehiscence	Lingual/Palatal fenestration
MAE	0.4663	0.6276	0.388	0.4451
RMSE	0.6170	0.7947	0.5428	0.5973
MRE	0.3008	0.3291	0.3550	0.3134
PCC	0.9722	0.9409	0.9480	0.9625

**FIGURE 5:**
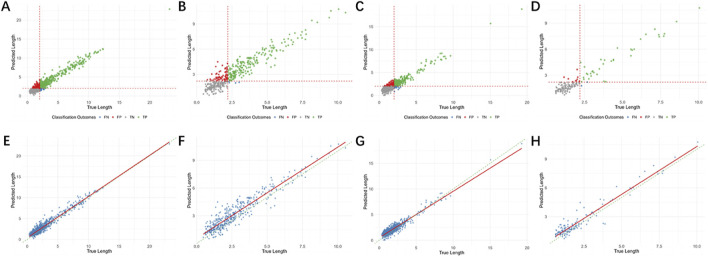
The distribution and comparison of predicted and true values for individual images. **(A**–**D)** Represent the distribution of predicted and true values. **(E**–**H)** Represent the comparison of true and predicted length. **(A) (E)** Labial/buccal dehiscence; **(B) (F)** Labial/buccal fenestration; **(C) (G)** Lingual/palatal dehiscence; **(D) (H)** Lingual/palatal fenestration.

**FIGURE 6:**
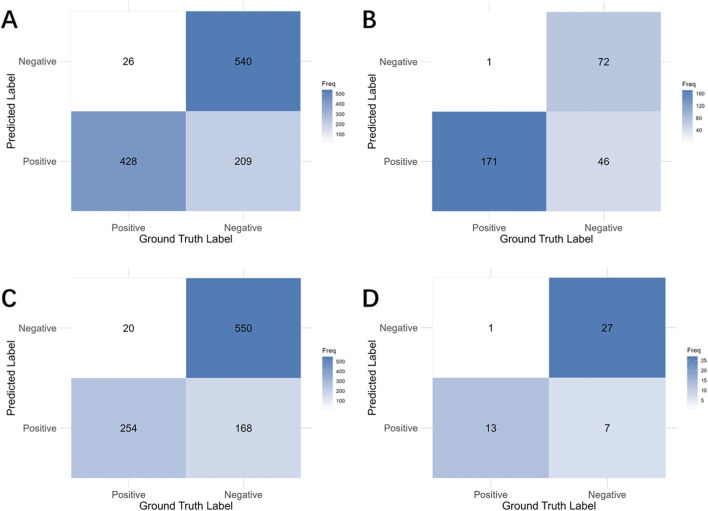
The confusion matrices for individual images. **(A)** Labial/buccal dehiscence. **(B)** Labial/buccal fenestration. **(C)** Lingual/palatal dehiscence. **(D)** Lingual/palatal fenestration.

**FIGURE 7:**
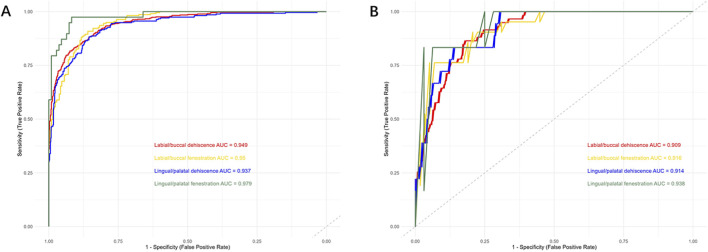
The ROC curves and AUC values. **(A)** Individual images. **(B)** Tooth-level diagnosis.

The performance metrics for individual image-based detection are presented in Table 4, while a distribution and comparison of predicted versus actual values are illustrated in Figure 5. For labial/buccal dehiscence, labial/buccal fenestration, lingual/palatal dehiscence, and lingual/palatal fenestration, the accuracy values were 0.8387, 0.8472, 0.8387, and 0.9257; Recall values were 0.9383, 0.9817, 0.9161, and 0.9487; and AUC values were 0.9485, 0.9505, 0.9368, and 0.9791, respectively. These results indicate high accuracy across all four sites and demonstrate high sensitivity for disease detection, confirming the robust performance of the individual image bone defect detection model. The corresponding confusion matrix is shown in Figure 6, and the ROC curves with AUC values are depicted in Figure 7A.

**TABLE 4:** Individual image disease detection performance.

Performance metric	Labial/Buccal dehiscence	Labial/Buccal fenestration	Lingual/Palatal dehiscence	Lingual/Palatal fenestration
Accuracy	0.8387	0.8472	0.8387	0.9257
Recall	0.9383	0.9817	0.9161	0.9487
Specificity	0.7784	0.7089	0.8092	0.9174
Precision	0.7196	0.7762	0.6469	0.8043
F1 score	0.8145	0.8669	0.7583	0.8706
AUC value	0.9485	0.9505	0.9368	0.9791

**FIGURE 8:**
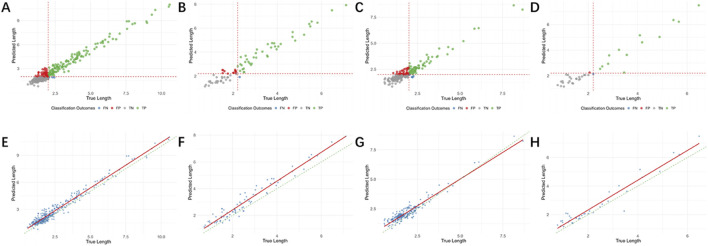
The distribution and comparison of predicted and true values for tooth-level diagnosis. **(A**–**D)** Represent the distribution of predicted and true values. **(E**–**H)** Represent the comparison of true and predicted length. **(A) (E)** Labial/buccal dehiscence; **(B) (F)** Labial/buccal fenestration; **(C) (G)** Lingual/palatal dehiscence; **(D) (H)** Lingual/palatal fenestration.

**FIGURE 9:**
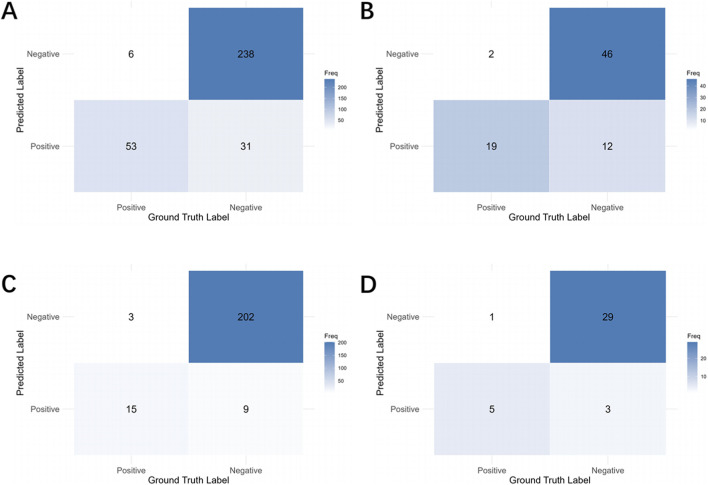
The confusion matrices for tooth-level diagnosis. **(A)** Labial/buccal dehiscence. **(B)** Labial/buccal fenestration. **(C)** Lingual/palatal dehiscence. **(D)** Lingual/palatal fenestration.

The performance metrics for tooth-level diagnosis, based on the consensus from three images, are summarized in Table 5. The distribution and comparison between predicted and actual values are shown in Figure 8. The respective accuracy values for the four defect types were 0.8872, 0.8228, 0.9476, and 0.8947; Recall values were 0.8983, 0.9048, 0.8333, and 0.8333; and AUC values were 0.9085, 0.9163, 0.9144, and 0.9375. The confusion matrix is presented in Figure 9, and the ROC curves with AUC values are provided in Figure 7B.

**TABLE 5:** Tooth-level diagnosis disease detection performance.

Performance metric	Labial/Buccal dehiscence	Labial/Buccal fenestration	Lingual/Palatal dehiscence	Lingual/Palatal fenestration
Accuracy	0.8872	0.8228	0.9476	0.8947
Recall	0.8983	0.9048	0.8333	0.8333
Specificity	0.8848	0.7931	0.9573	0.9062
Precision	0.6310	0.6129	0.6250	0.6250
F1 score	0.7413	0.7308	0.7143	0.7143
AUC value	0.9085	0.9163	0.9144	0.9375

## 4 Disccusion

### 4.1 Clinical significance and rationale for automated detection

Bone dehiscence and fenestration are prevalent forms of alveolar bone defects. Thorough evaluation of these defects is essential before orthodontic treatment to adjust force direction and magnitude, optimizing tooth movement to minimize risks and ensure long-term stability (Furlan et al., 2023). CBCT is the primary clinical tool for screening bone defects, though a standardized evaluation threshold for CBCT is lacking. In this study, we adopted the strictest threshold from existing literature: a bone defect at the alveolar crest exceeding 2 mm indicates bone dehiscence, while a continuity gap between the alveolar bone and root apex over 2.2 mm indicates bone fenestration (Sun et al., 2015).

### 4.2 Technical advantages of the swin UNETR model

Deep learning models have demonstrated promising potential for medical image analysis tasks, offering advantages such as high efficiency, accuracy, and usability compared to traditional manual interpretation by physicians (Dot et al., 2022; Schneider et al., 2022). In the oral field, classic CNN models have been successfully applied to automatically classify various dental and craniofacial structures, including jawbone density (Xiao et al., 2022), mid-palatal suture (Gao et al., 2022; Zhu et al., 2024), and lateral cephalometry (Yu et al., 2020). Furthermore, the CNN-transformer architecture UNet has been utilized for automated segmentation of dental CBCT images, showcasing the feasibility of deep learning models for segmenting tooth roots and alveolar bone (Chen et al., 2023). Building upon these advancements, the present study hypothesizes that deep learning models can be effectively employed to locate and measure bone defects. The complex anatomy of tooth roots and the similar radiographic density of these structures to surrounding bone tissue present challenges in the interpretation of CT images of periapical structures. Thin cortical bone layers often result in blurred imaging boundaries at the tooth root-bone interface. Furthermore, bone defects typically have a limited scope and irregular shapes. The combination of these factors leads to variability in interpretation results among clinicians with differing levels of experience. Prior to the Swin UNETR model, the research team utilized the U-net model for identifying bone defects, which struggled to accurately delineate the root-bone boundary. Although traditional CNN models can automatically segment teeth in CBCT images (Ayidh Alqahtani et al., 2023), they rely on local convolution kernels, limiting feature extraction to local regions. Even when the receptive field is expanded through stacked layers, the efficiency of modeling long-range dependencies remains limited. Consequently, these models are less effective at identifying low-contrast, blurred boundaries, and their ability to segment the root-bone boundary is constrained. Leveraging the innovative capabilities of the ViT in recent computer vision advancements (Chetoui and Akhloufi, 2022; Ko et al., 2024), the Swin UNETR model integrates skip connections with a CNN-based decoder, significantly enhancing semantic segmentation performance in medical imaging (Kakavand et al., 2025). This model captures long-range pixel dependencies across multiple resolutions, precisely identifies thin-layer bone interruptions, preserves the complete morphology of anatomical structures like the alveolar bone, and sharpens the delineation of bone defect edges. In this study, a heatmap is generated for the bone defect area, enabling the visualization of the disease and the quantitative diagnosis of bone dehiscence and fenestration.

### 4.3 Model performance and diagnostic efficacy

In this study, the Swin UNETR model achieves recognition rates of 92.97%–99.09% for dehiscence and fenestration, effectively ensuring comprehensive recognition of bone defects. The model demonstrated strong correlations between predicted and actual defect lengths, with PCC ranging from 0.9409 to 0.9722 across all four sites of dehiscence and fenestration. The model demonstrates precise positioning for bone fenestration and dehiscence, offering stable and reliable measurements that provide objective, quantitative imaging evidence for clinicians to assess disease severity and tailor treatment plans. Regarding disease detection performance, the accuracy ranges for the four sites were 0.8387–0.9257 (individual-image) and 0.8228–0.9476 (tooth-level). The corresponding recall ranges were 0.9161–0.9817 and 0.8333–0.9048, respectively. In contrast, precision was relatively lower, ranging from 0.6469 to 0.8043 for individual-image analysis and from 0.6129 to 0.6310 for tooth-level diagnosis. In disease discrimination, achieving high accuracy and recall is essential, with recall generally prioritized over precision. False positive results can be efficiently excluded through secondary review by clinicians. Conversely, missed diagnoses can compromise pre-operative risk prediction, potentially leading to complications like iatrogenic bone defects. Thus, this model exhibits outstanding performance in key metrics such as disease accuracy and overall discrimination efficacy, showing strong detection capabilities for bone dehiscence/fenestration diseases as well as high clinical relevance.

In the model analysis across the four regions, dehiscence accuracy is lower compared to fenestration. This discrepancy may be attributed to the higher frequency of dehiscence occurrences in clinical annotations relative to fenestration. Typically, a physiological distance exists between the alveolar crest and the CEJ, leading to a higher number of manual annotations for dehiscence. However, only defects exceeding 2 mm are classified as dehiscence. Consequently, the dataset predominantly comprises negative samples, given the limited positive annotations for fenestration. When the diagnostic model accurately identifies negative samples, the overall accuracy remains high even if its ability to identify positive samples is limited. Lingual/palatal fenestration, being the least common physiological bone defect, exhibits the highest accuracy. In this study, the average recognition and length error levels for fenestration exceed those for dehiscence. This discrepancy likely arises from the smaller sample size of physiological fenestration compared to dehiscence, reducing the model’s robustness and increasing error (Chang et al., 2023).

### 4.4 Clinical translation, limitations, and future directions

The Swin UNETR model aids in diagnosing and evaluating bone fenestration and dehiscence, significantly reducing clinicians’ manual measurement time. By automatically identifying defect boundary key points and calculating bone defect length, it eliminates measurement variability from subjective judgment, offering a quantitative basis for diagnosis. Conducting disease risk assessments prior to orthodontic treatment optimizes tooth movement paths and orthodontic force magnitude, enabling preemptive monitoring of high-risk tooth positions to prevent the worsening of iatrogenic bone defects (Choi et al., 2020). This study leverages artificial intelligence to enhance the accuracy and efficiency of clinical diagnoses in orthodontics, thereby reducing labor costs and improving the overall treatment process. Future implementations of deep learning and automated diagnostic programs in software could improve clinical communication between clinicians and patients.

This study has several limitations that warrant consideration for future research. First, the dataset consisted exclusively of CBCT sagittal images, excluding data from other planes. Clinicians are still required to manually locate the target tooth and extract its specific images within the CBCT data before submitting them to the model for analysis. Future advancements in high-resolution CBCT imaging and 3D reconstruction algorithms are expected to enable more precise lesion identification and annotation directly within the three-dimensional space. Furthermore, the current model provides a binary classification, indicating only the presence or absence of a bone defect without grading its severity. Subsequent studies should aim to expand the sample size to develop a more comprehensive diagnostic model capable of severity stratification.

## 5 Conclusion

This study successfully demonstrated the automated detection and quantitative measurement of bone fenestration and dehiscence in CBCT sagittal images using the SwinUNETR deep learning model. The model exhibited high accuracy across multiple tasks, including key point localization, defect length calculation, and disease classification. Notably, its high recall rate is particularly valuable for reducing the risk of missed diagnoses in clinical practice. This performance advantage stems from SwinUNETR’s architecture, which integrates a self-attention mechanism with an encoder-decoder structure, enabling it to effectively capture subtle features at the root-bone interface. However, this study has certain limitations. The analysis was confined to two-dimensional sagittal images and did not fully utilize three-dimensional spatial information. Furthermore, the data were sourced from a single medical center; thus, future validation using multi-center datasets is required to confirm its generalizability. The model demonstrates clear clinical translational potential as an auxiliary tool for pre-orthodontic risk assessment, promising to enhance diagnostic efficiency and consistency while reducing the subjectivity inherent in manual interpretation. Subsequent research will focus on developing a fully automated 3D bone defect detection system based on volumetric CBCT data. Additionally, we aim to explore the model’s application in disease severity stratification and long-term outcome prediction, thereby contributing to the advancement of intelligent diagnostics in dentistry.

## Data Availability

The original contributions presented in the study are included in the article/supplementary material, further inquiries can be directed to the corresponding author.
